# Multi-omics insight into muscle quality divergence between high-altitude Bayinbuluke sheep and low-altitude Turpan black sheep

**DOI:** 10.3389/fvets.2025.1682137

**Published:** 2025-11-07

**Authors:** Par Arshati Akhmiyati, Bin Chen, Yaling Yang, Lingling Liu, Wujun Liu

**Affiliations:** College of Animal Science, Xinjiang Agricultural University, Ürümqi, China

**Keywords:** Bayinbuluke sheep, metabolomics, proteomics, transcriptomics, Turpan black sheep

## Abstract

This study aimed to identify phenotypic biomarkers associated with high-altitude adaptation in Bayinbuluke sheep and to investigate the correlations between serum biochemical parameters and muscle transcriptomic, metabolomic, and proteomic profiles. Bayinbuluke sheep (raised at 3200 m) and Turpan black sheep (raised at−154 m) were selected for the experiment. The results demonstrated that, to adapt to the complex high-altitude hypoxic environment, Bayinbuluke sheep enhance glycolytic flux to rapidly generate energy, suppress intramuscular lipid synthesis, regulate lipid metabolic homeostasis to maintain energy balance, and remodel metabolic networks. Specifically, the GPAT3 gene promotes neutral cholesterol ester hydrolase 1 (NCEH1) through the glycerophospholipid metabolism pathway, facilitating the hydrolysis of cholesterol esters and fatty acid esters, thereby modulating systemic lipid metabolism. The FASN gene regulates energy metabolism via the AMPK signaling pathway, increasing the levels of glycolytic intermediates and markers such as nicotinamide adenine dinucleotide (NAD). Meanwhile, L-lactate dehydrogenase (LDHB) enhances the glycolytic process under hypoxic conditions through the HIF-1 signaling pathway, catalyzing the conversion between lactate and pyruvate in muscle tissue to produce energy, thereby supporting energy supply under high-altitude hypoxia. Additionally, the GSTA1 gene improves detoxification capability and antioxidant responses through the drug metabolism—other enzymes system, alleviating oxidative stress damage. This study systematically elucidates the molecular regulatory network underlying high-altitude adaptation in Bayinbuluke sheep, providing a theoretical foundation for enhancing the genetic adaptability of livestock resources in high-altitude environments.

## 1 Introduction

Lamb meat serves as an important source of high-quality protein, characterized by low cholesterol and fat content, and is rich in protein and vitamins. With evolving dietary consumption patterns, its contribution to total meat consumption in China has been gradually increasing (1). Bayinbuluke sheep, commonly referred to as Chateng big-tailed sheep, Bayinbuluke big-tailed sheep, or Bayinbuluke black-headed sheep, are primarily found in the Bayinbuluke Grassland of the Xinjiang Uygur Autonomous Region in northwestern China (2), with additional populations recorded in Hejing, Heshuo, Yanqi, Bohu, Luntai, and Korla (3). Turpan black sheep, also known as Turpan big-tailed black sheep, originated from long-term breeding and natural selection involving crosses among Bayinbuluke, Kazakh, and Karakul sheep in Tucksun County, Xinjiang Uygur Autonomous Region (4). Altitude is a critical factor influencing meat quality and has been shown to affect livestock meat traits through mechanisms such as hormone levels, fat deposition, and muscle metabolism (5). Studies suggest that mammals at high altitudes may generate energy through enhanced proteolytic and glycolytic pathways compared to those in lowland regions (6). However, the effect of altitude on the meat quality of Bayinbuluke and Turpan black sheep remains unexplored and warrants further investigation. Advances in molecular biology have established proteomics as a powerful tool for studying muscle biology (7). Proteomics involves the comprehensive identification and quantification of the expression, structure, function, localization, interactions, and post-translational modifications of all proteins within a cell, tissue, or organism at a specific time (8). Top-down proteomics (TDP), an emerging mass spectrometry (MS)-based technique, enables holistic characterization of intact protein forms (9). Metabolomics refers to the qualitative and quantitative analysis of low-molecular-weight metabolites across samples, linking differentially expressed metabolites to phenotypic variations and facilitating the identification of key metabolic drivers and preliminary mechanistic insights (10). It can elucidate interspecies interaction mechanisms and discover novel bioactive metabolites. Depending on the research objectives, metabolomics is categorized into untargeted (global, unbiased profiling) or targeted (focused quantification of specific metabolites) approaches (11). Serum biochemical parameters are widely recognized as indicators of metabolic and health status in animals (12). Evidence indicates that these parameters are significantly correlated with meat quality, influencing disease resistance, oxygen transport capacity, growth performance, genetic traits, and metabolic specificity (13). Previous research on Bayinbuluke sheep has focused solely on the effects of feeding regimens on meat quality (14), while studies on the impact of different altitudes remain unreported. This study integrates proteomic, metabolomic, transcriptomic, and serum biochemical analyses to systematically compare meat quality between Bayinbuluke sheep and Turpan black sheep. The aim is to uncover protein-level differences shaped by high- and low-altitude environments and to identify molecular targets for improved meat sheep breeding.

## 2 Materials and methods

### 2.1 Experimental animals and sample collection

This study employed 15 male Bayinbuluke sheep (BY group) and 15 male Turpan black sheep (TLF group), all 12 months of age and in good health, as experimental subjects. Prior to slaughter, all animals underwent a 24-h fast followed by a 2-h period of water deprivation. Body weight was recorded for each animal. Approximately 100 g samples were collected from three muscles: the longissimus dorsi (LD, at the level of the 12th–13th thoracic vertebrae), the triceps brachii (TB, lateral head at the mid-humerus), and the quadriceps femoris (QF, mid-portion of the rectus femoris). All samples were immediately snap-frozen in liquid nitrogen and stored at −80 °C for subsequent analysis.

### 2.2 Measurement indicators and methods

#### 2.2.1 Serum biochemistry analysis

Serum levels of the following biochemical parameters were determined using commercial ELISA kits (Nanjing Jiancheng Bioengineering Institute, China) in accordance with the manufacturer’s protocols: total protein (TP; g L^−1^), albumin (ALB; g L^−1^), superoxide dismutase (SOD; U mL^−1^), malondialdehyde (MDA; nmol mL^−1^), glutathione peroxidase (GSH-PX; U mL^−1^), catalase (CAT; U mL^−1^), total antioxidant capacity (T-AOC; mmol L^−1^), lactate dehydrogenase (LDH; U L^−1^), alanine aminotransferase (ALT; U L^−1^), creatinine (Cr; μmol L^−1^), uric acid (UA; μmol L^−1^), triglycerides (TG; mmol L^−1^), total cholesterol (TC; mmol L^−1^), calcium (Ca; mmol L^−1^), inorganic phosphate (Pi; mmol L^−1^), and immunoglobulin G (IgG; mg mL^−1^).

#### 2.2.2 Measurement of slaughtering performance

Measurement of pre-slaughter fasting live weight, carcass weight, and other relevant indicators was conducted. The specific procedure was as follows: the live weight of each sheep was measured before slaughter. Following slaughter and bleeding, the carcass was processed by removing the skin, hair, head, hooves, and internal organs, while retaining the kidneys. The remaining weight was recorded as the carcass weight. Subsequently, the slaughter rate was calculated based on the collected data using the following formula: slaughter rate (%) = (carcass weight of meat sheep / pre-slaughter fasting live weight of meat sheep) × 100.

#### 2.2.3 Determination of physical properties and nutrient content of meat

The carcass weight was recorded, and 100 g of muscle tissue samples were collected from each Bayinbuluke sheep (*n* = 15) and Turpan black sheep (*n* = 15) for subsequent analyses of meat physical properties and nutritional composition. Key measurements included shear force, moisture content, and other relevant parameters to evaluate meat quality attributes such as tenderness, pH, and color. All testing procedures were performed in accordance with the relevant Chinese national food safety standards (including but not limited to GB 5009.3–2016, GB 5009.5–2016, and GB 5009.6–2016). Mineral composition, including iron content, was determined based on appropriate national standards (e.g., GB 5009.90–2016, GB 5009.92–2016, GB 5009.93–2017, GB 5009.241–2017) (15).

### 2.3 Protein extraction

#### 2.3.1 Animal tissues

The samples were removed from −80 °C and ground into a fine powder in liquid nitrogen. Subsequently, an appropriate amount of the powdered sample was transferred to a 1.5 mL centrifuge tube, followed by the addition of lysis buffer (comprising 8 M urea, 1 mM PMSF, and 2 mM EDTA). The mixture was sonicated on ice for 5 min and then centrifuged at 15,000 g for 10 min at 4 °C to collect the supernatant. Finally, protein extraction was performed using the BCA kit (Shanghai Biyuntian Bio-Tech Co., Ltd.), and the protein concentration was determined.

#### 2.3.2 Protease desalting

A total of 100 μg of protein was aliquoted based on the quantified concentration, and the volume was adjusted to 200 μL with 8 M urea. Subsequently, dithiothreitol (DTT) was added to a final concentration of 5 mM, and the mixture was incubated at 37 °C for 45 min to reduce disulfide bonds. Alkylation was then conducted by adding iodoacetamide to a final concentration of 11 mM, and the solution was incubated in darkness at room temperature for 15 min. Next, 800 μL of 25 mM ammonium bicarbonate buffer and 2 μL of trypsin (Promega, V5280) were added, and enzymatic digestion was carried out overnight at 37 °C. After digestion, the peptide solution was acidified to pH 2–3 using 20% trifluoroacetic acid (TFA) and desalted using C18 stationary phase material (Millipore, Billerica, MA, USA). Finally, the concentration of the desalted peptide samples was determined using the Pierce™ Quantitative Peptide Detection Reagent with Standards kit (Thermo Fisher Scientific).

### 2.4 LC–MS/MS detection

#### 2.4.1 Nanolitre liquid chromatography detection

Samples obtained from Bayinbuluke sheep (*n* = 15) and Turpan black sheep (*n* = 15) were analyzed using a Vanquish Neo UHPLC nano-flow liquid chromatography system. The mobile phases consisted of 0.1% formic acid in water (A) and 0.1% formic acid in 100% acetonitrile (B). Separation was achieved using a trap-analytical dual-column configuration, which included a PepMap Neo Trap Cartridge (300 μm × 5 mm, 5 μm) and an Easy-Spray™ PepMap™ Neo UHPLC analytical column (150 μm × 15 cm, 2 μm). The analytical column was maintained at 55 °C via an integrated oven. A sample aliquot of 200 ng was loaded at a flow rate of 2.5 μL/min. The effective gradient duration was 6.9 min, with a total instrument run time of 8 min.

#### 2.4.2 Orbitrap astral mass spectrometer detection

Data-independent acquisition (DIA) mass spectrometry was performed using a nanoscale Vanquish Neo liquid chromatography system (Thermo Fisher Scientific) for chromatographic separation. Following nanoscale HPLC separation, samples were analyzed with an Orbitrap Astral high-resolution mass spectrometer (Thermo Scientific). The mass spectrometer was operated in positive ion mode with a precursor scanning range of 380–980 m/z. Full MS scans were acquired at a resolution of 240,000 (at 200 m/z), with a normalized automatic gain control (AGC) target of 500% and a maximum injection time of 5 ms. MS^2^ analysis was conducted in DIA mode using 299 scanning windows with an isolation window of 2 Th. Higher-energy collisional dissociation (HCD) was applied with a collision energy of 25%. The normalized AGC target for MS^2^ was set to 500%, with a maximum injection time of 3 ms (16).

### 2.5 RNA isolation and sequencing

#### 2.5.1 Total RNA extraction and quality control

Total RNA was isolated from the triceps brachii, longissimus dorsi, and quadriceps femoris muscles of Bayinbuluke sheep (*n* = 15) and Turpan black sheep (*n* = 15) using TRIzol reagent. RNA concentration and purity were determined using a NanoDrop 2000 spectrophotometer, while RNA integrity was assessed with an Agilent 2,100 Bioanalyzer using the RNA integrity number (RIN). Only samples with RIN values exceeding 8.5 and 28S/18S rRNA ratios above 0.7 were used for subsequent library preparation.

#### 2.5.2 Library preparation and sequencing

Total RNA was digested with DNase I to remove genomic DNA contamination, and mRNA was subsequently enriched using Oligo(dT)-coupled magnetic beads. The purified mRNA was fragmented, and double-stranded cDNA was synthesized and purified. Following end repair, adenylation, and adapter ligation, cDNA fragments of appropriate size were selected and amplified via PCR. The quality of the libraries was assessed based on concentration and insert size, with only those exceeding a final concentration of 2 nM being retained for sequencing. Paired-end sequencing (2 × 150 bp) was conducted on an Illumina NovaSeq 6,000 platform (Biomarker Technologies, Beijing, China).

#### 2.5.3 Quality control of raw sequencing data

The cDNA libraries constructed from RNA isolated from the triceps brachii, longissimus dorsi, and quadriceps femoris muscles of high-altitude Bayinbuluke sheep (*n* = 15) and low-altitude Turpan black sheep (*n* = 15) were sequenced on an Illumina high-throughput platform. Following adapter removal and quality control steps, high-quality clean reads were retained for subsequent bioinformatic analysis.

#### 2.5.4 Alignment to the reference genome

Clean reads were aligned to the *Ovis aries* reference genome (ARS-UI_Ramb_v3.0) using HISAT2 to generate accurate genomic mappings, including genomic distribution, alignment rate, and gene structural information. The aligned reads were then assembled into a reference-guided transcriptome using StringTie for subsequent downstream analyses.

### 2.6 Statistics and analyses

To comprehensively characterize protein function, data-independent acquisition mass spectrometry data were analyzed using DIA-NN (v1.8.1) against the UniProt *Ovis aries* proteome database (23,108 sequences), with identifications filtered at a false discovery rate (FDR) < 1%. Proteins exhibiting a fold change (FC) ≥ 1.5 or ≤ 0.667 and a *p*-value ≤ 0.05 were considered differentially abundant. Both these proteins and the full set of identifications were subjected to Kyoto Encyclopedia of Genes and Genomes (KEGG) pathway enrichment analysis based on a hypergeometric test, using all identified proteins as the background, to identify significantly enriched functional terms. Metabolomic datasets are high-dimensional and large in scale; thus, both univariate and multivariate statistical approaches were applied. Differential metabolites were screened using thresholds of variable importance in projection (VIP) > 1.0, FC > 1.2 or < 0.833, and *p* ≤ 0.05, with at least two biological replicates. Serum biochemical parameters were analyzed using independent-samples *t*-tests in SPSS Statistics 27.0 to assess statistical significance, and results are expressed as mean ± standard deviation.

## 3 Results

### 3.1 Serum biochemical indicators

Serum biochemical parameters of Bayinbuluke sheep (*n* = 15) and Turpan black sheep (*n* = 15) are summarized in Table 1. Levels of TP (g/L), ALB (g/L), SOD (U/mL), CAT (U/mL), T-AOC (mmol/L), LDH (U/L), Cr (μmol/L), UA (μmol/L), GLU (mmol/L), TG (mmol/L), and TC (mmol/L) were significantly higher in Bayinbuluke sheep compared to Turpan black sheep (*p* < 0.01). Additionally, MDA (nmol/mL), GSH-PX (U/mL), and ALT (U/L) were also notably elevated in Bayinbuluke sheep (*p* < 0.05). The remaining indicators showed no significant differences between the two breeds (*p* ≥ 0.05).

**Table 1:** Serum biochemical indices of Bayinbuluke and Turpan black sheep.

Items	BY	TLF
Protein TP (g/L)	54.31 ± 7.05**	38.63 ± 4.09
ALB (g/L)	28.59 ± 4.56**	19.21 ± 2.76
SOD (U/mL)	148.45 ± 22.50**	93.09 ± 10.80
GSH-PX (U/mL)	118.12 ± 124.03*	38.69 ± 7.20
CAT (U/mL)	3.50 ± 3.67**	0.79 ± 0.43
CK (U/mL)	0.44 ± 0.14^ns^	0.52 ± 0.15
ALP (King unit/100 mL)	35.43 ± 12.19^ns^	28.83 ± 8.44
ALT (U/L)	1.77 ± 0.59*	1.29 ± 0.36
UA (μmol/L)	94.55 ± 49.41**	45.71 ± 14.32
BUN (mmol/L)	3.44 ± 0.97^ns^	3.58 ± 0.73
T-BIL (μmol/L)	9.85 ± 3.74^ns^	7.85 ± 0.82
γ-GT (U/L)	72.45 ± 21.29^ns^	76.90 ± 16.30
GLU (mmol/L)	6.33 ± 1.43**	3.34 ± 1.01
TG (mmol/L)	0.41 ± 0.14**	0.24 ± 0.11
TC (mmol/L)	1.92 ± 0.54**	1.26 ± 0.24

For the same measure, data with a shoulder marker ** indicates a highly significant difference (*p* < 0.01), with a shoulder marker * indicates a significant difference (*p* < 0.05), and the abbreviation “ns” indicates a non-significant difference (*p* > 0.05).

### 3.2 Slaughter performance and physicochemical properties of muscle

#### 3.2.1 Refinement of slaughter performance

As presented in Table 2, no significant difference was observed in pre-slaughter live weight between the two breeds, averaging 35.74 kg for Bayinbuluke sheep (*n* = 15) and 32.65 kg for Turpan black sheep (*n* = 15). Similarly, carcass weight did not differ significantly, with values of 16.38 kg and 14.27 kg for Bayinbuluke and Turpan black sheep, respectively (*p* > 0.05). Furthermore, both slaughter rate and net meat rate were comparable between the two groups, with no statistically significant differences (*p* > 0.05).

**Table 2:** Results of slaughtering performance of Bayinbuluke sheep and Turpan black sheep.

Items	BY	TLF
Live-weight/kg	35.74 ± 3.91^ns^	32.65 ± 3.45
Carcass weight/kg	16.38 ± 2.24^ns^	14.27 ± 1.95
Slaughter rate	0.46 ± 0.03^ns^	0.44 ± 0.04

#### 3.2.2 Physicochemical properties of muscle

As presented in Table 3, the high-altitude Bayinbuluke sheep (*n* = 15) exhibited significantly greater moisture content, shear force, and iron concentration compared with the low-altitude Turpan Black sheep (*n* = 15) (*p* < 0.05). No significant differences were observed in the remaining indicators (*p* ≥ 0.05).

**Table 3:** Physicochemical properties of muscle of Bayinbuluke and Turpan black sheep.

Items	BY	TLF
Padding (%)	77.09 ± 0.41*	76.94 ± 0.75
Shearing force (kgf)	7.96 ± 2.29*	6.21 ± 1.51
Iron	16.41 ± 1.00**	15.76 ± 2.18

#### 3.2.3 Histological characteristics of muscle tissue

**Figure 1:**
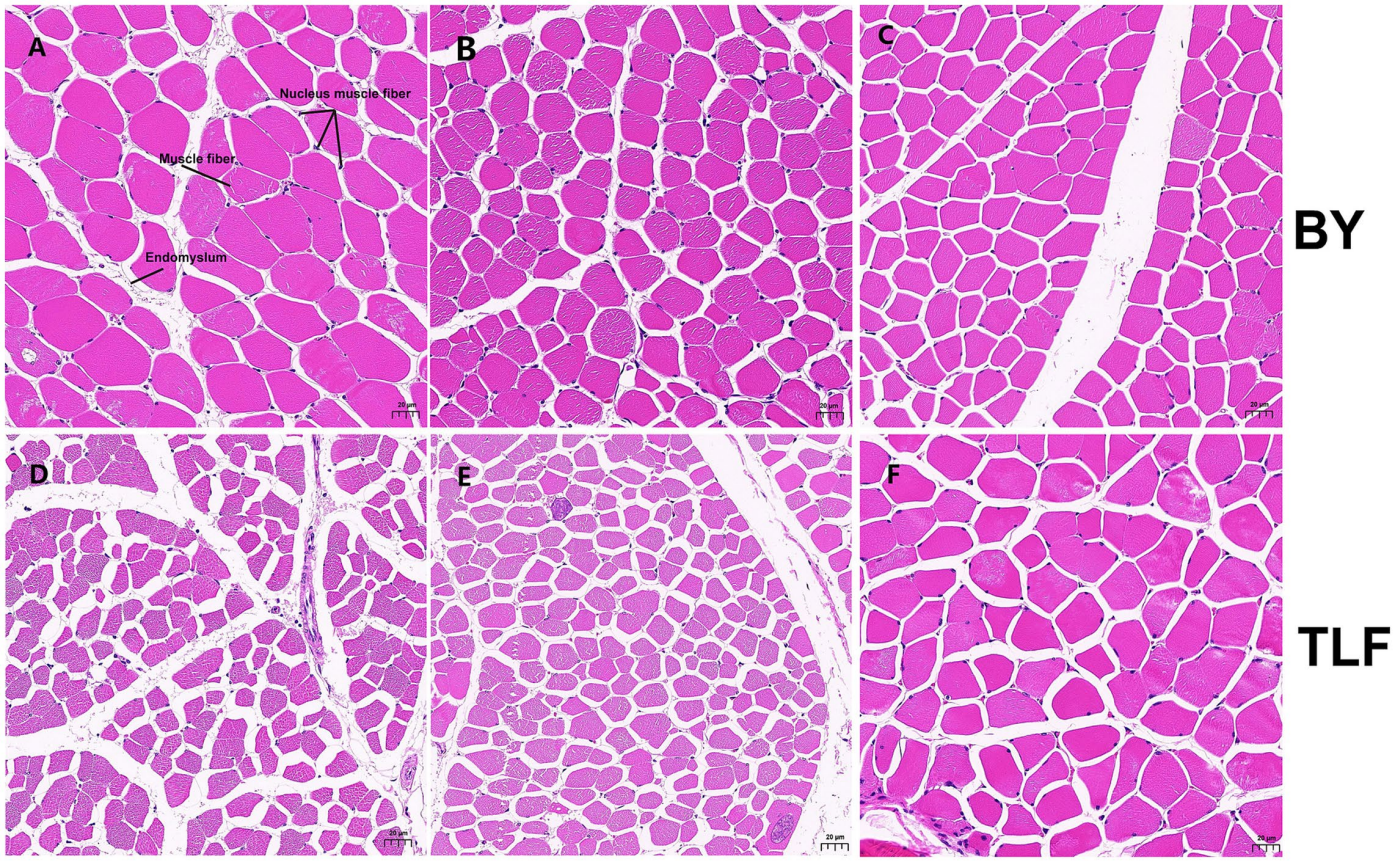
Muscle organization of Bayinbuluke and Turpan black sheep. **(A)** Triceps brachii muscle tissue section from a Bayinbuluke sheep; **(B)** Longissimus dorsi muscle tissue section from a Bayinbuluke sheep; **(C)** Quadriceps femoris muscle tissue section from a Bayinbuluke sheep; **(D)** Triceps brachii muscle tissue section from a Turpan black sheep; **(E)** Longissimus dorsi muscle tissue section from a Turpan black sheep; **(F)** Quadriceps femoris muscle tissue section from a Turpan black sheep.

As shown in Table 3, the high-altitude Bayinbuluke sheep (*n* = 15) demonstrated significantly higher moisture content, increased shear force, and elevated iron concentration compared to the low-altitude Turpan black sheep (*n* = 15) (*p* < 0.05). In contrast, no significant differences were observed in the remaining metrics between the two groups (*p* ≥ 0.05) (see Figure 1; Table 4).

**Table 4:** Detection results of muscle tissue related data of Bayinbuluke and Turpan black sheep.

Items	Laboratory animal	Organization		
		Longissimus dorsi	Triceps brachii	Quadriceps femoris muscles
Muscle fiber area	BY	1231.96 ± 511.85**	1605.78 ± 663.31**	1536.98 ± 522.63**
	TLF	775.91 ± 268.74	786.25 ± 251.87	1921.17 ± 797.90
Muscle fiber diameter	BY	35.15 ± 8.08**	41.39 ± 8.75**	38.77 ± 7.07**
	TLF	28.64 ± 5.15	27.67 ± 4.85	43.34 ± 9.63

For the same measure, data with a shoulder marker ** indicates a highly significant difference (*p* < 0.01), with a shoulder marker * indicates a significant difference (*p* < 0.05), and without a shoulder marker indicates a non-significant difference (*p* > 0.05). BY-B3: Triceps brachii of the Bayinbuluke sheep; TLF-B3: Triceps brachii of the Turpan black sheep; BY-G4: quadriceps femoris of the Bayinbuluke sheep, TLF-G4: quadriceps femoris of the Turpan black sheep; BY-BZ: longest dorsal muscle of the Bayinbuluke sheep, TLF-BZ: longest dorsal muscle of the Turpan black sheep, same as the following figure.

### 3.3 Proteomics

#### 3.3.1 Results of differential protein screening in muscle tissue

**Figure 2:**
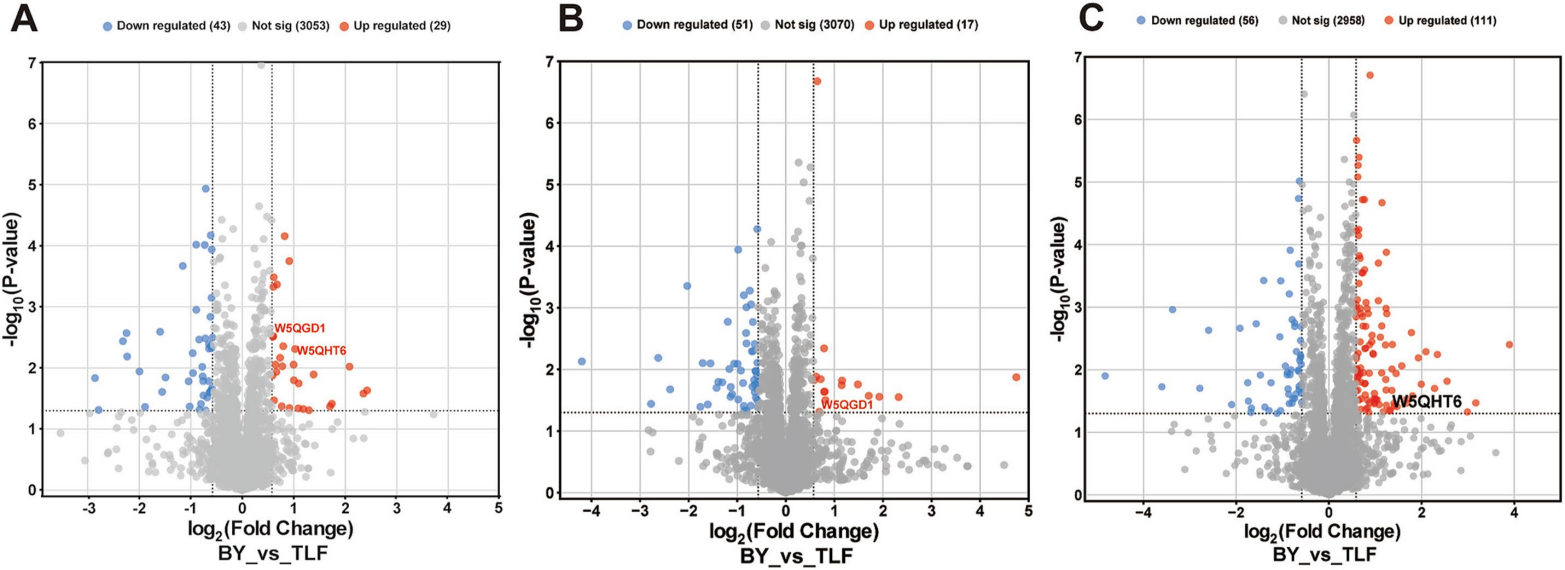
Volcano map of differential protein at different sites. **(A)** Volcano plot of differentially expressed proteins in BY-B3 vs. TLF-B3; **(B)** Volcano plot of differentially expressed proteins in BY-BZ vs. TLF-BZ; **(C)** Volcano plot of differentially expressed proteins in BY-G4 vs. TLF-G4. Differential protein volcano plots. Each dot represents an individual protein. Gray dots indicate proteins without significant differential expression, red dots represent significantly up-regulated proteins, and blue dots indicate significantly down-regulated proteins.

Volcano plots identified 29 up-regulated and 43 down-regulated differentially expressed proteins (DEPs) in the triceps brachii (Figure 2A), 17 up-regulated and 51 down-regulated in the longissimus dorsi (Figure 2B), and 111 up-regulated and 56 down-regulated in the quadriceps femoris (Figure 2C).

#### 3.3.2 KEGG pathway analysis of muscle tissue

**Figure 3:**
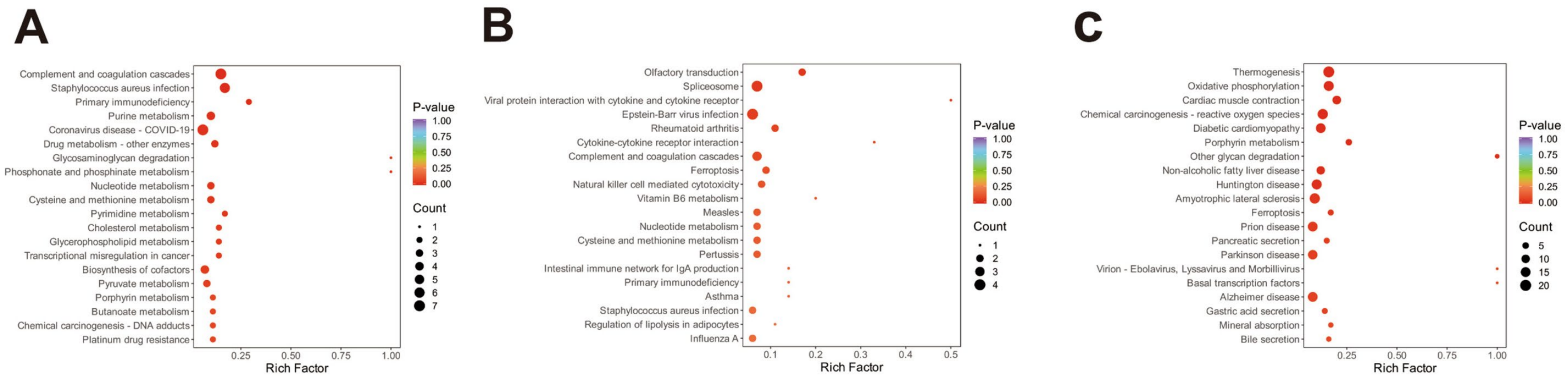
KEGG functional annotation analysis of the three sets of differentially expressed proteins. **(A)** Bubble plot of KEGG pathway enrichment analysis of differentially expressed proteins in BY-B3 vs. TLF-B3; **(B)** Bubble plot of KEGG pathway enrichment analysis of differentially expressed proteins in BY-BZ vs. TLF-BZ; **(C)** Bubble plot of KEGG pathway enrichment analysis of differentially expressed proteins in BY-G4 vs. TLF-G4. The bubble plot displays the KEGG pathway enrichment analysis of differentially expressed proteins. The size of the bubbles corresponds to the number of DEPs, while the color intensity indicates the level of statistical significance (darker red represents lower *Q*-value).

KEGG pathway enrichment analysis of DEPs was conducted to explore biological functions potentially related to ovine meat quality. In the comparison BY2-B3 vs. TLF2-B3 (Figure 3A), 52 DEPs were significantly enriched in 16 KEGG pathways (*p* < 0.05), four of which—cholesterol metabolism, glycerophospholipid metabolism, HIF-1 signaling, and AMPK signaling—were associated with lipid metabolism. In BY-BZ vs. TLF-BZ (Figure 3B), 11 DEPs were enriched in four pathways (*p* < 0.05), including AMPK signaling, ferroptosis, and the regulation of adipocyte lipolysis. For BY-G4 vs. TLF-G4 (Figure 3C), 166 DEPs were enriched in 14 pathways (*p* < 0.05), among which cholesterol metabolism was also linked to lipid metabolism. These pathways constitute major signaling and biochemical networks involving the identified DEPs.

### 3.4 Metabolomics

#### 3.4.1 Metabolomic differences in muscle tissue

**Figure 4:**
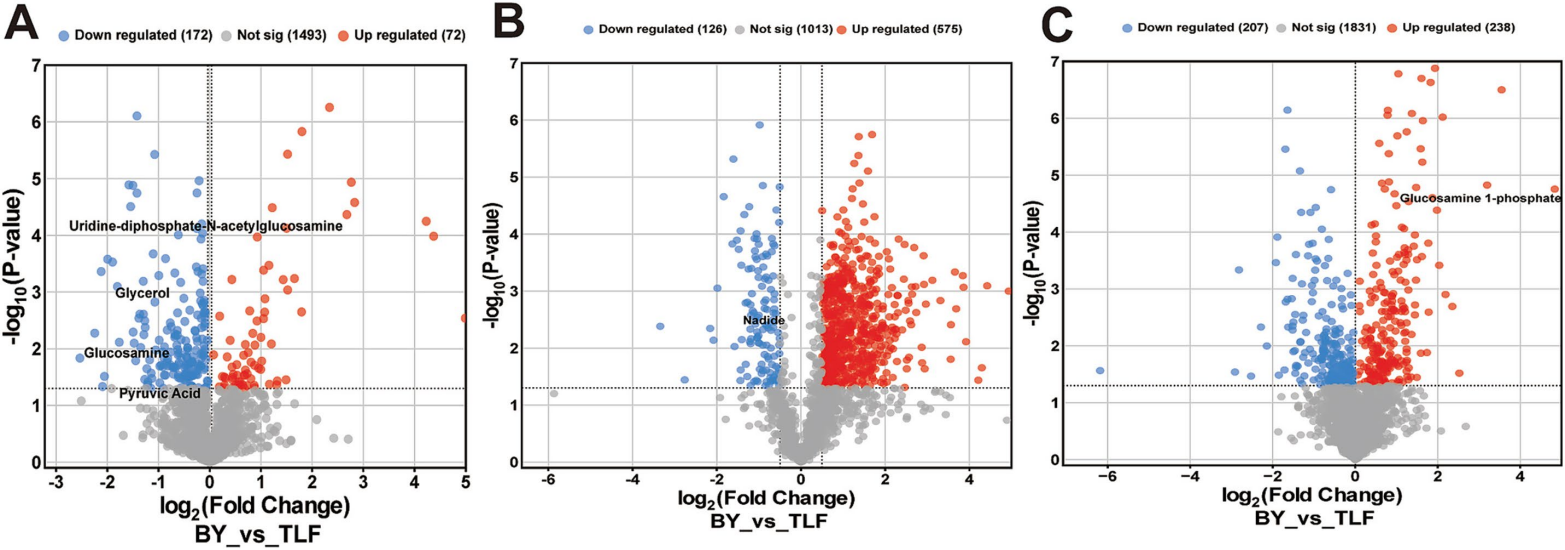
Volcano plot of differential metabolites among three muscle groups. **(A)** Volcano plot of differential metabolites in BY-B3 vs. TLF-B3; **(B)** Volcano plot of differential metabolites in BY-BZ vs. TLF-BZ; **(C)** Volcano plot of differential metabolites in BY-G4 vs. TLF-G4. Note: The *x*-axis represents the log2 fold change between comparison groups, and the *y*-axis indicates the level of statistical significance (−log10 *p*-value). Each point corresponds to an individual metabolite: red denotes up-regulated metabolites, blue indicates down-regulated metabolites, and gray represents metabolites with no significant differential expression.

Volcano plots (Figures 4A–C) identified 72 up-regulated and 172 down-regulated differential metabolites in the triceps brachii; 575 up-regulated and 126 down-regulated in the longissimus dorsi; and 238 up-regulated and 207 down-regulated in the quadriceps femoris.

#### 3.4.2 Analysis of KEGG pathways in muscle tissue

**Figure 5:**
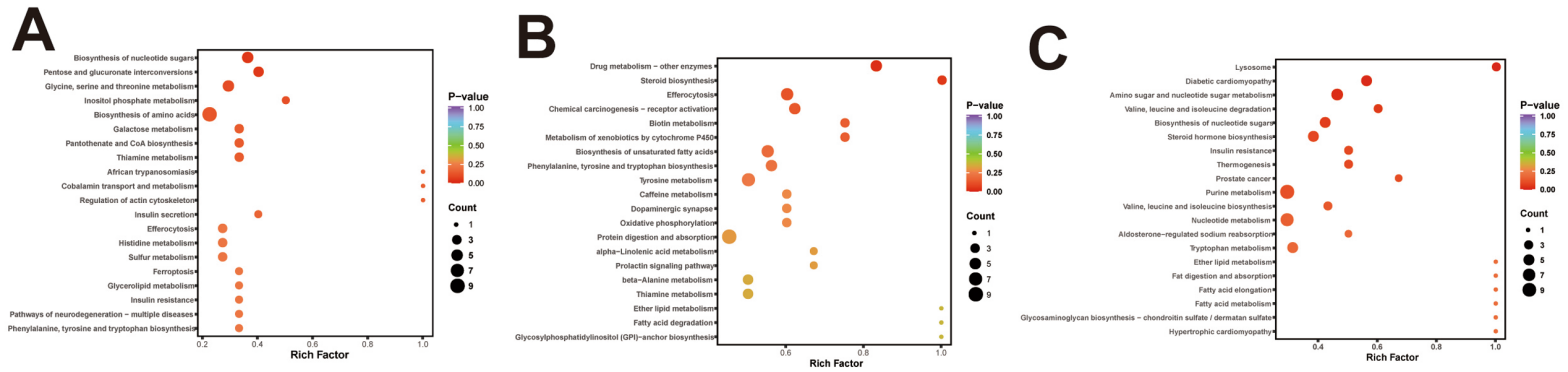
KEGG functional annotation analysis of differential metabolites in three muscle groups. **(A)** KEGG enrichment analysis of differentially expressed metabolites between BY-B3 and TLF-B3. **(B)** KEGG enrichment analysis of differentially expressed metabolites between BY-BZ and TLF-BZ. **(C)** KEGG enrichment analysis of differentially expressed metabolites between BY-G4 and TLF-G4. The bubble chart displays the KEGG pathway enrichment analysis of differentially expressed metabolites. The size of each bubble is proportional to the number of metabolites assigned to the pathway, and the color intensity indicates the level of statistical significance, with darker red representing lower *Q*-values.

KEGG pathway enrichment of differential metabolites was performed to elucidate their potential roles in ovine meat quality. As shown in Figure 5A, nine metabolites from the BY2-B3 versus TLF2-B3 comparison were significantly enriched (*p* < 0.05) in the nucleotide-sugar biosynthesis and pentose–glucuronate interconversion pathways, both of which govern sugar and energy metabolism essential for myogenesis and meat formation. In the BY-BZ versus TLF-BZ comparison, five metabolites were significantly enriched (*p* < 0.05) in one pathway associated with xenobiotic detoxification rather than with direct meat-quality traits (Figure 5B). Six metabolites from the BY-G4 versus TLF-G4 comparison were significantly enriched (*p* < 0.05) in three pathways: amino sugar and nucleotide sugar metabolism, diabetic cardiomyopathy, and lysosomal function (Figure 5C). Among these, the amino sugar and nucleotide sugar metabolism pathway mirrors the molecular profile underlying meat formation, whereas alterations in lysosomal activity regulate myofibrillar protein turnover and consequently affect meat maturation.

### 3.5 Transcriptomics

#### 3.5.1 Transcriptomic differences in muscle tissue

**Figure 6:**
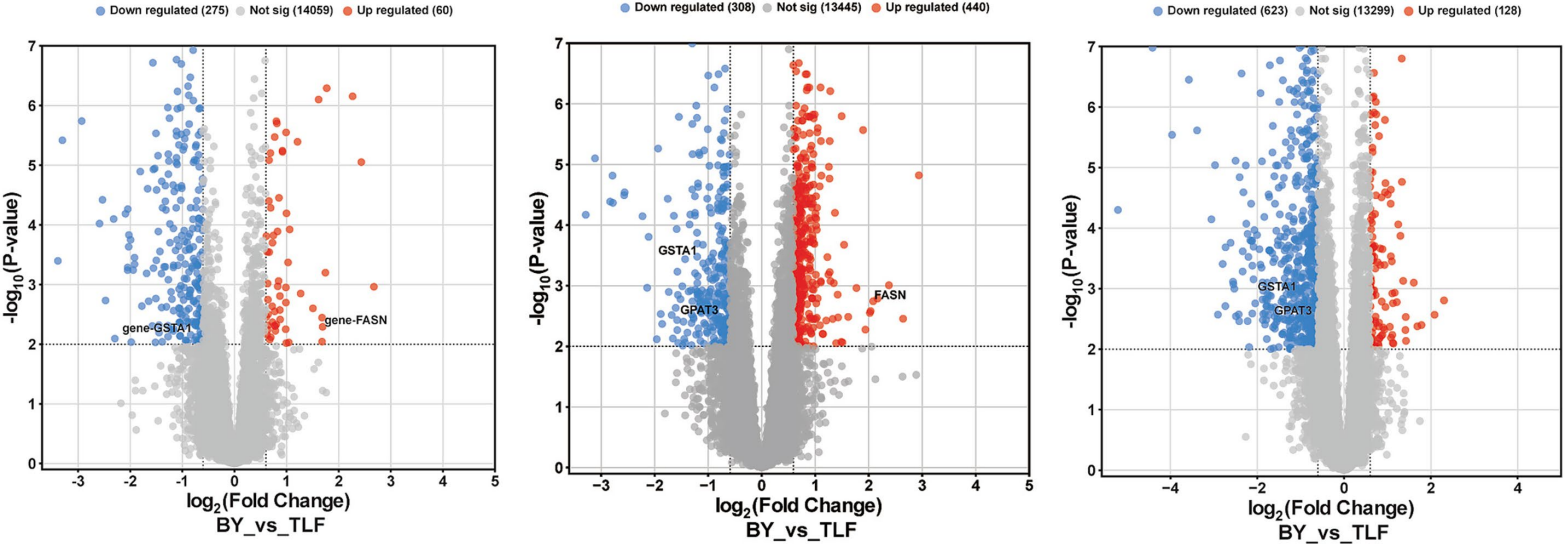
Volcano plots of differentially expressed genes in various conditions. **(A)** Volcano plot of differentially expressed genes between BY-B3 and TLF-B3. **(B)** Volcano plot of differentially expressed genes between BY-BZ and TLF-BZ. **(C)** Volcano plot of differentially expressed genes between BY-G4 and TLF-G4. Volcano plots visualize differences in gene expression between groups. The *x*-axis represents the log₂(fold change), and the *y*-axis corresponds to the −log10(p-value), indicating the magnitude and statistical significance of expression changes, respectively. Each point denotes a single gene: red indicates up-regulated DEGs, blue indicates down-regulated DEGs, and gray represents genes with no significant differential expression.

Differentially expressed genes (DEGs) were defined using thresholds of |fold-change| ≥ 1.5 and FDR < 0.01 (Figures 6A–C). In the triceps brachii, 337 DEGs were identified, including 62 up-regulated and 275 down-regulated genes. In the longissimus dorsi, 748 DEGs were detected, of which 440 were up-regulated and 308 down-regulated. In the quadriceps femoris, 751 DEGs were identified, comprising 128 up-regulated and 623 down-regulated genes.

#### 3.5.2 KEGG enrichment analysis of muscle tissue

**Figure 7:**
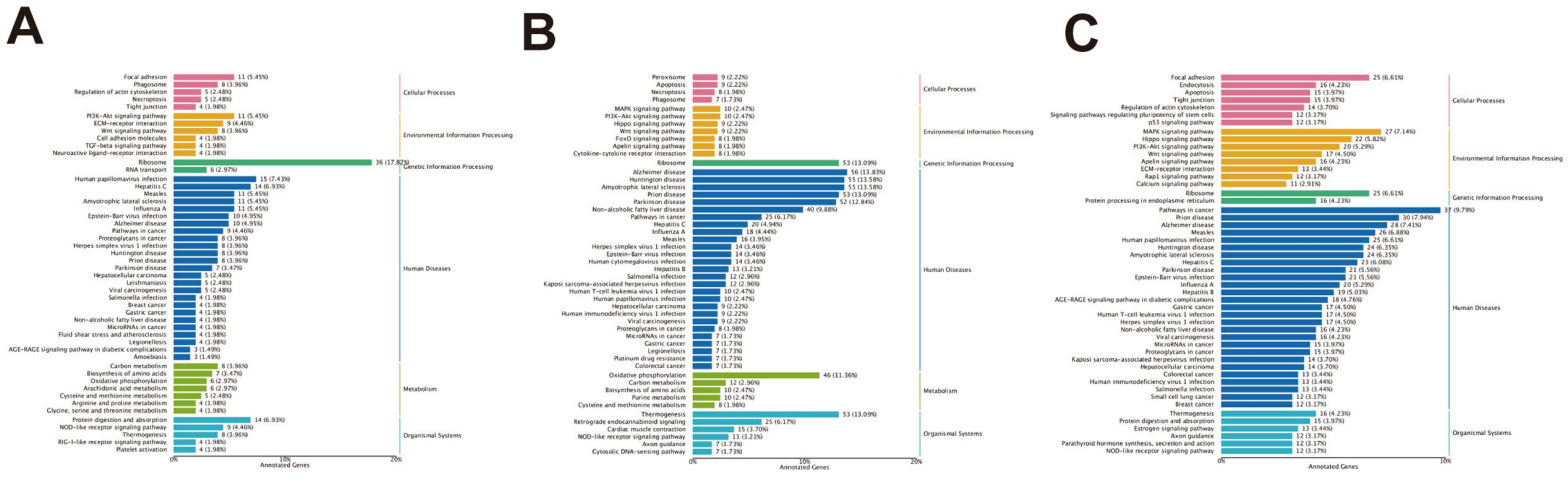
Functional annotation analysis of differentially expressed genes in KEGG for different parts. **(A)** KEGG enrichment analysis of differentially expressed genes between BY-B3 and TLF-B3. **(B)** KEGG enrichment analysis of differentially expressed genes between BY-BZ and TLF-BZ. **(C)** KEGG enrichment analysis of differentially expressed genes between BY-G4 and TLF-G4. Note: The bar chart displays the KEGG pathway enrichment analysis of differentially expressed genes. The length of each bar corresponds to the ratio of differentially expressed genes enriched in a specific pathway to the total number of KEGG-annotated DEGs. Longer bars indicate a higher degree of enrichment. The color gradient represents the range of *p*-values, with more intense colors denoting greater statistical significance.

KEGG pathway enrichment analysis was conducted to investigate the differentially expressed genes between high-altitude Bayinbuluke sheep and low-altitude Turpan black sheep. In the comparison BY2-B3 vs. TLF2-B3 (Figure 7A), 337 genes were significantly enriched in 199 pathways (*p* < 0.05), among which five pathways were related to lipid metabolism: drug metabolism – other enzymes, HIF-1 signaling, AMPK signaling, pentose and glucuronate interconversions, and glycerophospholipid metabolism. For BY-BZ vs. TLF-BZ (Figure 7B), 748 genes were assigned to 285 significantly enriched pathways (*p* < 0.05), including cholesterol metabolism, amino sugar and nucleotide sugar metabolism, HIF-1 signaling, glycerophospholipid metabolism, AMPK signaling, drug metabolism – other enzymes, and pentose and glucuronate interconversions. In the BY-G4 vs. TLF-G4 comparison (Figure 7C), 751 genes were enriched in 250 pathways (*p* < 0.05), with lipid metabolism-related pathways such as drug metabolism—other enzymes, glycerophospholipid metabolism, amino sugar and nucleotide sugar metabolism, and AMPK signaling. These pathways represent key biochemical and signaling networks associated with the differentially expressed genes.

#### 3.5.3 Integrated analysis of multi-omics data

**Figure 8:**
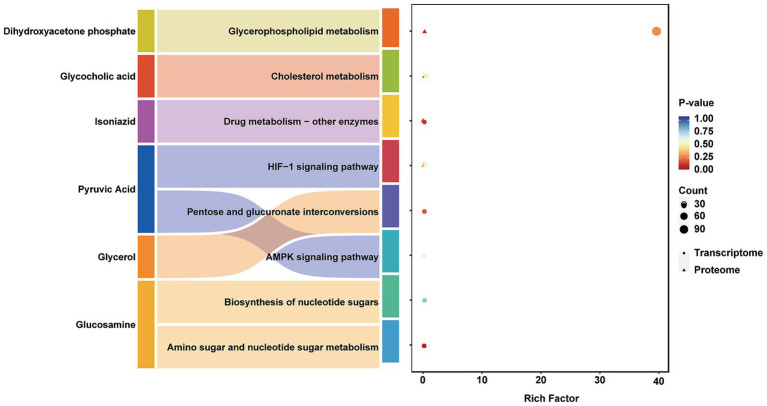
Integrated analysis of proteomics, metabolomics and transcriptomics. Circular dots represent transcriptomic pathways, while triangular markers denote proteomic pathways. The *Y*-axis displays pathway names, and the *X*-axis represents the Rich Factor, defined as the ratio of the number of differentially expressed molecules enriched in a given pathway to the total number of background molecules associated with that pathway. The size of each marker corresponds to the number of differentially expressed molecules enriched in the pathway, and the color indicates the *p*-value from the enrichment analysis, with a gradient from red to blue representing increasing significance.

Differential analysis was performed separately on the metabolomic, proteomic, and transcriptomic datasets. Significantly differentially expressed genes and proteins from transcriptomic and proteomic data were identified using a standard threshold (*p* < 0.05), while significant metabolites were selected based on VIP > 1.0 and *p* < 0.05. KEGG pathway enrichment analysis was subsequently conducted for all significantly altered molecules. To identify biological pathways commonly regulated across omics layers, pathways significantly enriched (*p* < 0.05) in at least two omics datasets were extracted and defined as multi-omics intersection pathways. These cross-omics pathways were visualized using a bubble plot (Figure 8). This analytical approach enabled clear identification of core pathways—including glycerophospholipid metabolism, cholesterol metabolism, and the AMPK signaling pathway—that were significantly perturbed across multiple omics levels. These pathways collectively form a multi-layered regulatory network within muscle tissue, demonstrating considerable biological significance.

## 4 Discussion

### 4.1 Serum biochemical indicators

Serum biochemical indicators reflect the health and metabolic status of animals and are closely correlated with nutrient uptake and metabolism (17). This study compared serum biochemical profiles of high-altitude Bayinbuluke sheep and low-altitude Turpan black sheep to elucidate breed differences in meat-quality traits. Total protein (TP) and albumin (ALB) maintain plasma osmolality and pH; their serum levels indicate hepatic protein-synthetic capacity and overall nutritional status (18). In this study, the TP content (g/L) and ALB content (g/L) were found to be significantly higher in Bayinbuluke sheep compared to Turpan black sheep. It is hypothesized that this differential expression of total protein may contribute to a greater ability for muscle protein deposition in Bayinbuluke sheep. This phenomenon could be linked to more active liver function, increased efficiency of protein anabolism, or superior nutrient uptake, thereby indirectly regulating muscle tissue growth and development.

The serum glucose (GLU) level (mmol/L) serves as an indicator of the energy metabolism within the animal organism, representing a primary substrate for ATP production and supplying the necessary energy required by the host (19). Elevated GLU levels are indicative of increased energy metabolism (20). Research has demonstrated that GLU functions as an essential energy substrate that sustains tissue and organ functionality, facilitating energy provision to the body through enhanced glycolysis, thereby maintaining ATP levels (21). In this experiment, it was observed that the serum glucose GLU content (mmol/L) in Bayinbuluke sheep was significantly higher than that in Turpan black sheep. This finding suggests that elevated energy metabolism in the muscle tissues of Bayinbuluke sheep may promote glycolytic processes, consequently enhancing ATP production efficiency within these muscles. This observation aligns with previous findings discussed above.

Total cholesterol (TC) and triglycerides (TG) are crucial lipid components in the blood. TG serves as the primary form of energy storage in the body, playing a significant role in energy metabolism, while TC is an essential precursor for cell membrane composition and the synthesis of steroid hormones and vitamin D. Studies have demonstrated that lipids are metabolized by the liver into TC and TG, which subsequently enter circulation by binding to low-density lipoproteins (22). High-density lipoprotein (HDL) facilitates the transport of TC deposited within the inner walls of blood vessels back to the liver for lipid metabolism, thereby preventing atherosclerosis (23). In this study, serum total cholesterol levels (mmol/L) and triglyceride concentrations (mmol/L) were found to be significantly higher in Bayinbuluke sheep compared to Turpan black sheep. It is hypothesized that Bayinbuluke sheep possess a more active capacity for lipid metabolism within their muscles, which may be associated with their enhanced energy reserves and utilization efficiency.

Catalase (CAT) (U/mL), glutathione peroxidase (GSH-PX) (U/mL), and superoxide dismutase (SOD) (U/mL) are the primary antioxidant enzymes in organisms, which effectively scavenge free radicals (24). These enzymes play a crucial role in the antioxidant defense mechanisms of living organisms. In this study, serum levels of CAT and SOD were found to be significantly higher in Bayinbuluke sheep compared to Turpan black sheep. Additionally, the content of GSH-PX was also significantly elevated in Bayinbuluke sheep relative to Turpan black sheep across all groups. Bayinbuluke sheep displayed significantly higher serum CAT, SOD, and GSH-PX activities (*p* < 0.05), denoting a robust antioxidant capacity. This enhanced system is capable of effectively scavenging reactive oxygen species (ROS), thereby mitigating oxidative stress-related damage to muscle cells.

### 4.2 The physicochemical characteristics of meat

Moisture and other essential components of lamb significantly influence the juiciness of the meat, with moisture content being a direct determinant. Water-holding capacity is one of the critical factors affecting both tenderness and juiciness in meat products. Most of the water present in meat is stored within myogenic fibers, muscle fibers, and their interstitial spaces (25). Research has demonstrated that elevated moisture content significantly influences meat quality, particularly in terms of tenderness and juiciness. Typically, higher moisture levels lead to softer and juicier meat, while also impacting meat color and water retention properties (26). These findings align with those observed in the current experimental study. Jin et al. (27) demonstrated that Merganser’s forelegs possess a denser musculature along with an enhanced ability to retain water. van Laack et al. (28) established that intramuscular fat content influences muscle shear; specifically, higher intramuscular fat levels correlate with reduced muscle shear force. In this study, the shear force of Bayinbuluke sheep was significantly greater than that of Turpan black sheep (*p* < 0.05), which we hypothesize is closely related to lower fat deposition levels. Reduced fat deposition leads to diminished intermuscular fat content—this component plays a buffering role within muscle tissue and can decrease friction between muscle fibers, thereby influencing shear force. Therefore, low fat deposition combined with reduced intermuscular fat content and tightly structured muscle fibers may serve as significant contributors to the elevated shear force observed in Bayinbuluke sheep. Additionally, iron content was found to be significantly higher compared to Turpan black sheep (*p* < 0.01). As a key component of myoglobin, iron enhances colour stability and antioxidant capacity, thereby improving meat quality (29).

### 4.3 Differential expression characteristics of the muscle tissue proteome

In skeletal muscle of Bayinbuluke and Turpan black sheep, the NCEH1 protein is enriched in the glycerophospholipid metabolism and cholesterol metabolism pathways; LDHB is enriched in the HIF-1 signaling pathway. The cholesterol metabolism pathway and the glycerophospholipid metabolism pathway are related to lipid metabolism, while the HIF-1 signaling pathway is associated with energy metabolism.

Neutral cholesterol ester hydrolase 1 (NCEH1), as a crucial serine hydrolase, is mainly distributed in adipose tissue and liver cells. It participates in lipid catabolism by catalyzing the hydrolysis of cholesterol esters and fatty acid esters (30). In a diabetic mouse model, Sun et al. (31) observed that NCEH1 deficiency diminished cholesterol ester synthesis and consequently impaired endothelial function, establishing NCEH1 as a critical regulator of cholesterol ester metabolism. Moreover, Matsuoka et al. (32) demonstrated that retinoic acid receptor-related orphan receptor-*α* (RORα) promotes cholesterol ester hydrolysis in macrophages by directly activating NCEH1 transcription, thereby reducing lipid droplet accumulation. In the present study, up-regulation of NCEH1 is postulated to modulate intramuscular lipid deposition and distribution via its regulatory role in lipid metabolism, thereby influencing meat tenderness, juiciness, and flavor.

L-lactate dehydrogenase (LDHB) catalyses the reversible conversion of pyruvate to lactate during glycolysis and is particularly active under hypoxia, maintaining systemic pH (33). Kim et al. (34) reported that increasing the activity of lactate dehydrogenase is conducive to the generation of NADH, which is used for the reduction of metmyoglobin to maintain the stability of meat color. LDHB converts lactic acid into pyruvic acid to maintain its growth and metabolism (35). In this study, we observed that the expression of LDHB in the muscle tissue of Bayinbuluke sheep was significantly upregulated. By promoting glycolysis, maintaining muscle pH and NADH supply, it enhanced the stability of meat color and affected the accumulation of flavor amino acids, thereby improving the flavor and color of the meat.

### 4.4 Differential expression characteristics of the metabolome in muscle tissue

In the muscle tissues of Bayinbuluke sheep and Turpan black sheep, glucosamine and uridine diphosphate-N-acetylglucosamine were enriched in the biosynthesis pathway of nucleotide sugars, while glycerol and pyruvate were enriched in the interconversion pathway of pentose and glucuronic acid; nicotinamide adenine dinucleotide was significantly enriched in the drug metabolism - other enzyme systems pathway; and glucosamine 1-phosphate was significantly enriched in the amino sugar and nucleotide sugar metabolism pathway.

Glucosamine is a precursor of uridine diphosphate-N-acetylglucosamine, which plays a significant role in glycosylation modifications and glycan synthesis (36). In animal hydrolysates, glucosamine has been identified as having potential for flavor modulation (37). This indicates that glucosamine and its derivatives may influence the flavor and texture of meat by affecting the glycosylation modifications of proteins, thereby impacting the overall sensory characteristics of the meat.

Glycerol is an important biochemical substance that is widely present in microorganisms. Within cells, glycerol is synthesized and decomposed through multiple metabolic pathways, playing a crucial role in providing energy and carbon sources (38). Glycerol serves as one of the primary substrates in phospholipid biosynthesis and, along with dihydroxyacetone phosphate (DHAP), acts as an intermediate in glycolysis (39). In muscle metabolism, glycerol is converted into pyruvate via the glycolytic pathway. This pyruvate then enters the mitochondria, where it undergoes complete oxidation and decomposition into carbon dioxide and water, thereby releasing energy necessary for vital bodily functions (40). This process not only provides energy to muscles but also plays a role in fat metabolism, which influences both the flavor and tenderness of muscle tissue.

Pyruvic acid, also known as *α*-oxopropionic acid, is a crucial intermediate in the sugar metabolism of all living cells and plays a significant role in the interconversion of various substances within the body. Its metabolic flux is closely associated with the synthesis of flavor precursors, such as nucleotides and amino acids (41). As an end product of glycolysis, pyruvate exhibits antioxidant properties (42). Studies have demonstrated that pyruvate can inhibit the oxidation of oxygen free radicals in rats (43). In this study, we observed down-regulation of pyruvate expression; being an end product of glycolysis, its reduced levels may inhibit mitochondrial oxidative phosphorylation and subsequently affect ATP production. This inhibition could lead to insufficient energy supply for muscle contraction and repair capacity in muscle fibers, ultimately resulting in decreased tenderness (44).

Nicotinamide adenine dinucleotide (NAD) is a crucial coenzyme in cellular energy metabolism and plays a significant role in various redox reactions (45). The involvement of NAD in glycolysis and the tricarboxylic acid cycle is vital for proper muscle metabolism and energy supply (46). Yang et al. (47) demonstrated that exogenous supplementation of NAD^+^ and NADH to yak *M. longissimus* thoracis significantly elevated AMPK activity and accelerated post-mortem glycolysis during aging. This intervention enhanced the rate of energy metabolism and up-regulated AMPK protein expression, indicating that NAD^+^ promotes energy turnover in stored muscle by activating AMPK and concomitantly increases muscle pH (47). Additionally, both NAD and its metabolite NADH influence meat flavor and texture through their participation in metabolic processes related to energy production.

Glucosamine 1-phosphate is a phosphorylated derivative of glucosamine that plays a significant role in the metabolism of amino sugars. Research has demonstrated that glucosamine can enhance sweetness perception by inducing the Maillard reaction, while also exerting specific effects on umami and egg yolk flavors (48). The presence of this metabolite in meat may indirectly influence its flavor and texture through its participation in the Maillard reaction.

### 4.5 The interplay between serum biochemical indicators and multi-omics

In this study, Bayinbuluke sheep exhibited a higher level of liver metabolism, enhanced antioxidant enzyme activity, and improved protein metabolism capabilities. The differences observed in proteomic expression indicated that the expression of LDHB was down-regulated, leading to reduced fat deposition. This suggests that Bayinbuluke sheep may have developed a unique adaptation mode characterized by “high metabolism-low fat deposition.” The up-regulation of NCEH1 expression influenced fat deposition and intramuscular fat distribution through the regulation of lipid metabolism. Additionally, PFKFB4-mediated modulation of the AMPK signaling pathway and involvement of LDHB in glycolysis further optimized intramuscular fat distribution, energy metabolism, and pH stability. Metabolomics analysis revealed that the levels of differential metabolites such as uridine 5′-diphosphate-N-acetylgalactosamine were down-regulated in the muscles of Bayinbuluke sheep, which impacted energy metabolism and glycosylation modifications. This down-regulation has multiple implications for normal muscle function and overall health. Conversely, the up-regulation of certain differential metabolites like glucosamine 1-phosphate promotes the accumulation of flavor compounds. By participating in amino sugar metabolic processes, these metabolites influence meat qualities such as flavor, taste, tenderness, and juiciness. Transcriptomic studies indicate that the expression level of the differentially expressed gene FASN is upregulated, enhancing the synthesis of long-chain fatty acids (49) and regulating fat deposition in the body (50) to adapt to high-altitude hypoxic environments. Conversely, the GSTA1 gene exhibits downregulation; it reduces cellular apoptosis by inhibiting oxidative stress, thereby protecting muscle fibers (51). Similarly, GPAT3 also shows a downward trend in expression. This gene decreases triglyceride (TAG) synthesis (52) while accelerating fatty acid (FA) oxidation, leading to reduced lipid accumulation in the liver (53), while simultaneously promoting systemic fat deposition (54). This process facilitates “hepatic-muscle lipid flux redistribution. Through these combined effects, the meat quality characteristics of Bayinbuluke sheep include lower fat deposition, more stable meat color, enhanced accumulation of flavorful amino acids, superior tenderness, and juiciness.

## 5 Conclusion

In this study, we investigated the serum biochemical phenotypes, muscle sections, proteomics, metabolomics and transcriptomics of Bayinbuluke and Turpan black sheep to elucidate the significance of lipid transport, energy metabolism, and antioxidant capacity in meat quality. Through the analysis of serum biochemical indices, it was found that total protein (TP) and glucose (GLU) are directly associated with lipid metabolism and energy metabolism. Additionally, catalase (CAT) and glutathione peroxidase (GSH-PX) were identified as key indicators related to oxidative stress resistance. Proteomic analysis revealed an up-regulation in the expression of neutral cholesterol ester hydrolase 1 (NCEH1) and phosphofructokinase-2/fructose-2,6-bisphosphatase 4 (PFKFB4), which play crucial roles in regulating pathways linked to lipid metabolism. Conversely, lactate dehydrogenase B (LDHB) expression was down-regulated to mitigate excessive fat accumulation and reduce fatty acid synthesis. Metabolomic analysis indicated a down-regulation in six differential metabolites—including uridine 5′-diphosphate-N-acetylgalactosamine, glycerol, and pyruvate—affecting energy metabolism regulation. Furthermore, glucosamine 1-phosphate along with 1-methyl-4-nitroimidazole exhibited up-regulated expression that influenced meat flavor and taste. Transcriptomic analysis indicates that the upregulation of FASN gene expression enhances fat deposition in the body through lipid metabolism-related pathways. Concurrently, the downregulation of GSTA1 and GPAT3 gene expressions synergistically inhibits oxidative stress and blocks hepatic triglyceride synthesis, thereby promoting the directed deposition of fatty acids into skeletal muscle. These findings further complement existing research on the meat quality traits of Bayinbuluke sheep and provide new data support for subsequent investigations into its potential biological mechanisms.

## Data Availability

The data supporting the findings of this study are available in the following repositories: [Figshare] under http://doi.org/10.6084/m9.figshare.29551862; [National Center for Biotechnology Information] under accession number PRJNA1306583 (http://www.ncbi.nlm.nih.gov/bioproject/1306583); and [Integrated Proteome Resources] under reference number IPX0012591000 (https://www.iprox.cn/).
